# Efficacy of Mindfulness‐Based Interventions for Reducing Cancer‐Related Fatigue: A Systematic Review and Meta‐Analysis

**DOI:** 10.1002/pon.70435

**Published:** 2026-03-23

**Authors:** Jihyun Lee, Myoungsuk Kim

**Affiliations:** ^1^ College of Nursing Kangwon National University Chuncheon‐si Gangwon‐do Republic of Korea

**Keywords:** cancer, fatigue, meta‐analysis, mindfulness‐based intervention

## Abstract

**Background:**

Cancer‐related fatigue (CRF) is a common and distressing symptom among patients with cancer. Although mindfulness‐based interventions (MBIs) are increasingly applied for CRF management, evidence regarding their efficacy remains inconsistent.

**Aims:**

This systematic review and meta‐analysis aimed to evaluate the efficacy of MBIs in reducing CRF.

**Methods:**

PubMed, EMBASE, Web of Science, Cochrane, and CINAHL were comprehensively searched from database inception to August 31, 2025, for randomized controlled trials (RCTs) involving patients with cancer who received MBIs. The primary outcome of interest was fatigue. The risk of bias was assessed using the Cochrane risk‐of‐bias 2.0 tool. A meta‐analysis was performed using a random‐effects model, and standardized mean differences (SMDs) with 95% confidence intervals (CI) were calculated. Subgroup analyses, meta‐regressions, publication bias assessments, and sensitivity analyses were also performed.

**Results:**

A total of 29 RCTs involving 3178 participants were included. Compared with control interventions, MBIs were associated with significantly lower fatigue levels (SMD = −0.89; 95% CI: −1.23 to −0.55, *p* < 0.001). However, substantial heterogeneity was observed (I^2^ = 87.3%). Sample size significantly moderated the results. Although publication bias was detected, the adjusted effect size remained significant. Sensitivity analysis confirmed the robustness of the findings.

**Conclusions:**

This study demonstrates that MBIs are an efficacious approach for significantly reducing fatigue among patients with cancer, supporting the role and broader application of nonpharmacological strategies in managing CRF. However, substantial between‐study heterogeneity, moderate certainty of evidence, and indications of publication bias warrant cautious interpretation of the pooled estimates.

## 1 Background

Despite the increasing number of patients with cancer, substantial progress has been made in early detection and treatment, leading to improved survival outcomes [1]. However, many cancer survivors experience various physical and psychological symptoms that adversely affect their quality of life, including cancer‐related fatigue (CRF), pain, sleep problems, and emotional distress [2]. Among these, CRF can occur at any stage of cancer treatment [2, 3]. The National Comprehensive Cancer Network (2023) defines CRF as “a distressing, persistent, and subjective sense of tiredness or exhaustion related to cancer or cancer treatment that is disproportionate to recent activity and is not fully relieved by rest or sleep.” [4] Unlike ordinary fatigue, CRF depletes physical energy, impairs cognitive function, destabilizes emotions, and hinders social functioning, thereby reducing treatment adherence and overall quality of life [2, 4].

Approximately 60%–90% of patients with cancer experience CRF during or after treatment, and in some cases, symptoms persist chronically [5]. As CRF is influenced by physical, emotional, and cognitive factors, it is challenging to manage with a single intervention, which highlights the need for holistic nursing strategies [2, 4]. Pharmacologic treatments, including antidepressants, psychostimulants, and corticosteroids, aim to address the physiological mechanisms underlying CRF. However, their use in clinical settings remains limited because of variable efficacy and concerns regarding side effects [6]. Consequently, nonpharmacologic interventions, including exercise, cognitive‐behavioral therapy (CBT), psychoeducation, and relaxation training, have been explored [5, 7]. Although exercise can reduce fatigue, it may be challenging for patients with physical limitations [5, 8]. Similarly, CBT can alleviate fatigue‐related distress, but it requires professional guidance and substantial time, limiting its practicality in routine care [7, 9].

For these reasons, mindfulness‐based interventions (MBIs), which integrate physical, psychological, and emotional dimensions of well‐being, have gained increasing attention as nonpharmacologic approaches to symptom management among patients with cancer [10, 11]. Importantly, MBIs are not a single, uniform intervention; rather, they represent a heterogeneous family of programs unified by a shared therapeutic focus on cultivating nonjudgmental awareness of present‐moment experiences [12]. While the specific content, delivery format, and intensity of these programs vary, they are theorized to engage common underlying psychological processes, such as attentional regulation, heightened body awareness, and reduced emotional reactivity. Within oncology settings, MBIs have been associated with significant reductions in anxiety, depression, sleep disturbances, and pain while also enhancing positive affect and overall quality of life [13, 14]. Viewed through this lens, MBIs may alleviate the subjective experience of CRF by shifting a patient's attentional focus, fostering a more accepting relationship with bodily sensations, and mitigating maladaptive stress responses [11].

Despite these benefits, evidence regarding the effectiveness of MBIs in CRF management remains inconclusive. Although several previous meta‐analyses have examined the efficacy of MBIs in alleviating CRF, these studies have methodological limitations, particularly in study populations and design. Some studies included only female patients with cancer [15, 16], limiting the generalizability of their findings. Others incorporated nonrandomized controlled trials (non‐RCTs) [13, 17], thereby reducing the reliability of the results. Additionally, some studies focused exclusively on mindfulness‐based stress reduction (MBSR) programs [16, 18], making it difficult to comprehensively evaluate other mindfulness intervention modules. Moreover, some studies [13, 19, 20, 21, 22] included a small number of trials, which limited statistical power and the validity of their conclusions. To address these limitations, this study systematically and comprehensively evaluated the efficacy of MBIs in improving CRF by including diverse cancer patient populations and various types of interventions while synthesizing findings exclusively from rigorous randomized controlled trials (RCTs). Specifically, it aimed to address the following research question: *To what extent do MBIs alleviate cancer‐related fatigue?*

By addressing this question, the study attempts to provide a comprehensive synthesis of current high‐quality evidence and clarify MBIs' potential to mitigate CRF across diverse clinical contexts.

## 2 Methods

This systematic review and meta‐analysis was conducted in accordance with the Cochrane Handbook for Systematic Reviews of Interventions (version 6.5)^23^ and reported following the Preferred Reporting Items for Systematic Reviews and Meta‐Analyses (PRISMA) 2020 guidelines. The protocol was prospectively registered in PROSPERO (registration number: CRD420251112897) to minimize bias and enhance reproducibility.

### 2.1 Information Sources and Search Strategy

PubMed, EMBASE, Web of Science, CENTRAL, and CINAHL Plus were comprehensively searched for literature from database inception to August 31, 2025. The search strategy was developed based on the Population, Interventions, Comparisons, Outcomes, and Study Design (PICOSD) framework and the recommendations of the Cochrane Collaboration. The strategy incorporated a combination of Medical Subject Headings (MeSH), free‐text terms, and Boolean operators. The primary keywords used in the search included combinations of (neoplasms OR carcinoma OR tumor OR cancer OR Oncol*) AND (mindful* OR mindfulness‐based* OR mindfulness OR MBSR OR MBCR OR MBCT OR body mind) AND (fatigue OR cancer‐related fatigue OR lassitude) AND (randomized controlled trials). No language restrictions were applied. In addition, the reference lists of all included studies were manually screened to identify additional relevant publications. Detailed search strategies for all databases are available in Supporting Information S1: Tables S1–S5.

### 2.2 Eligibility Criteria

The inclusion and exclusion criteria were defined according to the PICOSD framework as follows: (1) Population (P): Adults (≥ 18 years) diagnosed with any type of cancer; (2) Intervention (I): MBIs delivered as structured programs over a specified period; (3) Comparison (C): Usual care, waitlist control, routine care, basic education, or no intervention. Studies were also included if the active control condition involved general cancer‐related education or fatigue management education not based on mindfulness; (4) Outcomes (O): CRF was assessed using validated and reliable self‐report instruments; (5) Study Design (SD): Only RCTs were included to ensure methodological rigor and minimize potential bias.

Studies were excluded if they met any of the following conditions: (1) Participants were patients receiving pharmacologic treatment for depression or anxiety or were diagnosed with psychiatric disorders (e.g., schizophrenia, bipolar disorder) or severe cognitive impairment (e.g., moderate‐to‐severe dementia); (2) The experimental group received combined interventions in which MBIs were implemented alongside other therapies (e.g., relaxation, music, or aromatherapy) or an MBI was delivered as a single‐session intervention; (3) The control group received mindfulness or similar interventions (e.g., meditation, yoga, relaxation training), or the study lacked a control group (i.e., single‐group design); (4) The study did not report sufficient statistical data (e.g., means and standard deviations) for meta‐analysis or failed to provide pre‐ or post‐intervention data; (5) The study design was not an RCT, such as a quasi‐experimental, observational, or survey‐based research; and (6) Full text was not available (e.g., abstract only, study protocol, unpublished manuscript).

### 2.3 Study Selection

All retrieved records were imported into EndNote 20 (Clarivate Analytics, Philadelphia, PA, USA) for reference management and duplicate removal. The study selection process was conducted in two phases. In the first phase, two reviewers (MK and JL) independently screened the titles and abstracts of all identified studies to exclude irrelevant articles. Discrepancies at this stage were resolved through discussions between both reviewers until consensus was reached. In the second phase, the full texts of the remaining articles were thoroughly reviewed to determine their eligibility according to the predefined PICOSD criteria. Each reviewer independently assessed the studies to minimize selection bias. Disagreements regarding study inclusion were resolved through discussion and consensus between the reviewers.

### 2.4 Data Extraction

To ensure both accuracy and consistency, two reviewers (MK and JL) independently extracted data, and discrepancies were resolved through detailed discussions until consensus was achieved. The following data were extracted: study characteristics (author, year, country); clinical characteristics (cancer stage and type, treatment status, sample size, mean age, and percentage of female participants); intervention features (program type, delivery method, instructor background, and intervention duration); measurement instrument; primary outcome; control group; and adverse events. Standardized mean differences (SMDs) were calculated as post‐intervention minus pre‐intervention values, with post‐intervention values obtained from the time point closest to the end of the intervention period. Missing data were identified during extraction. When alternative statistics were available, they were converted into means and standard deviations using Cochrane‐recommended formulas. If no suitable data were provided, attempts were made to contact the corresponding authors. Studies for which the necessary information could not be obtained were excluded from the meta‐analysis.

### 2.5 Risk of Bias Assessment

The risk of bias for each RCT was independently evaluated by two reviewers (MK and JL) using the Cochrane Risk of Bias 2.0 tool [24]. The tool examines five key domains: the randomization process, deviations from intended interventions, missing outcome data, measurement of outcomes, and selection of reported results. Each domain is rated as having “low risk,” “some concerns,” or “high risk” of bias. The reviewers compared their individual assessments and resolved inconsistencies through collaborative discussions until consensus was reached. Subsequently, an overall risk of bias judgment was assigned to each study, categorized as follows: (1) When all domains were rated as having a low risk of bias, the overall risk was considered “low”; (2) when at least one domain was assessed as having a high risk of bias, the overall risk was deemed “high”; and (3) when some domains were rated as having “some concerns” or “low risk,” the overall risk of bias was classified as “some concerns.”

### 2.6 Certainty of Evidence Assessment

The certainty of the evidence was independently assessed by two reviewers (MK and JL) following the Grading of Recommendations, Assessment, Development, and Evaluations (GRADE) approach [25] using the GRADEpro software (McMaster University and Evidence Prime Inc., Hamilton, Canada). The evaluation considered five domains: risk of bias, inconsistency, indirectness, imprecision, and publication bias. The overall certainty of the evidence was classified into four categories: “high,” “moderate,” “low,” and “very low.” Any discrepancies between the reviewers were resolved through discussion until consensus was achieved.

### 2.7 Synthesis and Statistical Analysis

A meta‐analysis was conducted using the *meta* package in R (version 4.5.1; R Foundation for Statistical Computing, Vienna, Austria). A random‐effects model was employed to account for heterogeneity across studies. This approach assumes that each study may have a unique effect size and is appropriate for capturing differences in population characteristics or intervention methods [26]. Because different outcome measures were used across studies, SMDs with 95% confidence intervals (CIs) were calculated to compare effect sizes [23]. Studies with lower standard errors were assigned greater weights using the inverse variance method, reflecting their higher precision [27]. Effect sizes were categorized as small (SMD, 0.2 to < 0.5), moderate (SMD, 0.5 to < 0.8), or large (SMD, ≥ 0.8), consistent with standard meta‐analysis guidelines [28].

Heterogeneity among studies was evaluated using the I^2^ statistic, which measures the percentage of total variation due to true heterogeneity rather than chance. Values between 0% and 40% were interpreted as “not important; ” 30% and 60%, as “potentially moderate heterogeneity; ” 50% and 90%, as “substantial heterogeneity; ” and 75% and 100%, as “considerable heterogeneity.” [23] When substantial heterogeneity was detected, moderator analyses were performed to identify the sources. Subgroup analyses were conducted for categorical moderators (e.g., cancer type, treatment status, program type, intervention duration, delivery method, instructor background). When differences in effect sizes between groups were significant, their statistical significance was evaluated using analysis of variance within the meta‐analytic model. For continuous moderators (e.g., mean age, sample size, percentage of female participants), random‐effects meta‐regression analyses were conducted to examine the relationships between moderators and effect sizes.

To assess publication bias, funnel plot symmetry was visually inspected, followed by Egger's regression test for statistical verification. Publication bias was indicated by a *p*‐value of < 0.05. When publication bias was detected, the trim‐and‐fill method was applied to adjust for potentially missing studies and evaluate its impact on the results. Finally, a sensitivity analysis was conducted to assess the robustness of the meta‐analysis findings.

## 3 Results

### 3.1 Selected Studies

**FIGURE 1:**
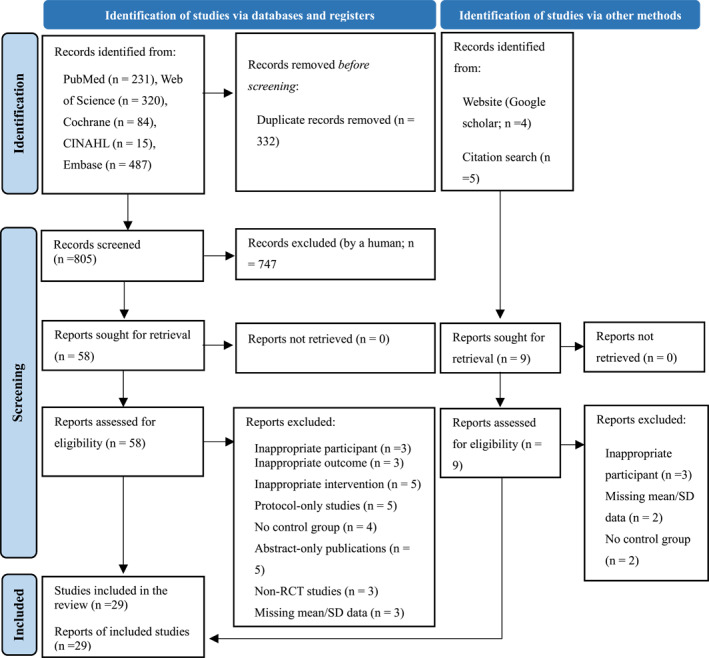
Flowchart summarizing the process of study selection.

The initial search identified a total of 1137 articles. After removing 332 duplicates, 805 articles remained. Following title and abstract screening, 747 articles were excluded, leaving 58 articles for full‐text review. Of these, 31 articles were excluded for the following reasons: inappropriate population (*n* = 3), inappropriate outcomes (*n* = 3), inappropriate interventions (*n* = 5), protocol‐only studies (*n* = 5), no control group (*n* = 4), abstract‐only publications (*n* = 5), non‐RCT studies (*n* = 3), and missing mean/SD data (*n* = 3) (Figure 1).

### 3.2 Characteristics of the Included Studies

The most common study setting was the United States (*n* = 12 studies) [14, 29, 30, 31, 32, 33, 34, 35, 36, 37, 38, 39], followed by China (*n* = 6)^40‐45^ and Iran (*n* = 4) [46, 47, 48, 49]. Studies were also conducted in Canada (*n* = 1) [50], Japan (*n* = 1) [51], the Netherlands (*n* = 1) [52], the Republic of Korea (*n* = 1) [53], Switzerland (*n* = 1) [54], Turkey (*n* = 1) [55], and the United Kingdom (*n* = 1) [56]. Cancer stages ranged from 0 to IV; three studies [29, 34, 49] did not report staging information. Breast cancer was the most frequently studied cancer type (*n* = 18), followed by other cancer types (*n* = 9). Treatment status varied widely, including patients currently undergoing treatment and those who had completed treatment. A total of 3178 cancer patients were included, with sample sizes ranging from 12 to 167 participants in the intervention groups and 8 to 155 participants in the control groups. The mean age of patients was 51.25 years; three studies did not report the mean age. The majority of participants (93.1%) were female.

MBSR was the most common intervention program (*n* = 15). The majority of the interventions were delivered in person (group sessions) (*n* = 23), most of them were implemented online (via the web or an application) (*n* = 4) or through a combination of in‐person and online delivery (*n* = 2). The most frequent intervention duration was 8 weeks (*n* = 16), followed by 6 weeks (*n* = 7). The most commonly used fatigue assessment tool was the Fatigue Symptom Inventory (*n* = 8), followed by the Brief Fatigue Inventory (*n* = 4). Eleven studies measured fatigue as the primary outcome. Regarding control conditions, waitlist controls were the most common (*n* = 13), followed by usual care controls (*n* = 8). Adverse events were rarely reported. Eight studies explicitly reported that none of the participants experienced adverse events. Only 1 study reported mild knee pain (1.4%) and temporary lower extremity weakness (1.4%), but no serious adverse events were observed (Table 1).

**TABLE 1:** Characteristics of the randomized controlled trial studies (*N* = 29).

No.	Author (Year), Country	Cancer stage	Cancer type	Treatment status	Sample size		Mean age ± SD	Female (%) (E/C)	Program type/delivery method/instructor	Intervention duration (sessions per week, h)	Instrument	Primary outcome	Comparison	Adverse events
					E	C	E/C							
1	Basharpoor (2025), Iran [46]	I‐III	Various cancer	Completed all treatments, except for hormonal therapy, at least 3 months prior	15	15	44.33 ± 7.88/42.86 ± 4.0	100/100	MBCR/group sessions/trained staff	8 weeks (1/week, 1.5 h)	CFS	Yes	Usual care	NR
2	Blaes (2016), USA [29]	NR	Various cancer	Completed cancer treatment	26	8	55 ± 10/57 ± 10	93/86	MBCR/group sessions/trained faculty	8 weeks (1/week, 2.5 h)	FACIT‐F	Yes	Wait‐list	NR
3	Bower (2015), USA [30]	0‐III	BC	Completed cancer treatment (except hormone therapy)	39	32	46.1 (range 28.4–60)/47.7 (range 31.1–59.6)	100/100	MAPs/group sessions/NR	6 weeks (1/week, 2 h)	FSI	No	Wait‐list	NR
4	Bower (2021), USA [31]	0‐III	BC	Completed cancer treatment	85	81	44.5 ± 7.7/45.9 ± 5.6	100/100	MAPs/group sessions/certified instructors	6 weeks (1/week, 2 h)	FSI	No	Wait‐list	None
5	Chu (2020), China [40]	0‐III	BC	Underwent breast cancer surgery	42	42	54.6 ± 5.7/54.9 ± 6.3	100/100	MBCT/group session/CR, psychiatrists, nurses	8 weeks (1/week, 2 h and one 6h session)	BFI	No	General psychological counseling and routine care	NR
6	Enjezab (2024), Iran [47]	I‐III	BC	≥ 1‐month post‐diagnosis, ≤ 2 months post‐RT/CT, or pre‐treatment	28	28	47.57 ± 9.36/51.86 ± 10.75	100/100	MBSR/online (WhatsApp)/instructor with a background in nursing and midwifery	8 weeks (1/week, 2 h)	FSS	Yes	Wait list	NR
7	Grossman (2015), Switzerland [54]	0‐II	Various	Completion HSCT, complete remission	33	29	52.1 ± 14.1	Both group: 50	MBI/group sessions + AOMC/certified instructors	8 weeks (1/week, 2.5 h and one 6 h session)	FACIT‐F	No	AOMC (four individual telephone consultations)	NR
8	Gu (2024), China [41]	I‐IV	Cervical cancer	Newly diagnosed with cervical cancer	40	38	NR	100/100	MBSR/online (WechatApp)/NR	8 weeks	CFS	Yes	Wait‐list	NR
9	Hoffman (2012), UK [56]	0‐III	BC	Completion of surgery or CT or RT	114	115	49.0 ± 9.26/50.1 ± 9.14	100/100	MBSR/group sessions/clinician–researcher	8 weeks (1/week, 2 h, and one 6 h session)	POMS	No	Wait‐list	None
10	Jang (2016), Republic of Korea [53]	0‐III	BC	Completion of surgery or CT or RT	12	12	51.75 ± 5.32/51.42 ± 6.33	100/100	MBAT/group sessions/Mental health medicine specialist	12 weeks (1/week, 45 min)	EORTC‐QLQ‐C30	No	Wait‐list	NR
11	Janusek (2019), USA [32]	0‐III	BC	Newly diagnosed with early‐stage BC	96	96	55.0 ± 10.1/55.2 ± 10.1	100/100	MBSR/group sessions/CR	8 weeks (1/week, 2.5 h and one 6 h session)	MFSI‐SF	No	ACC (general education))	None
12	Johns (2015), USA [33]	I‐IV	Various (BC 83.3%)	Completed CT/RT	18	17	58.8 ± 9.3/55.7 ± 9.3	94/94	MBSR/group sessions/certified instructors	7 weeks (1/week, 2 h)	FSI	Yes	Wait‐list	None
13	Johns (2016), USA [14]	0‐III	BC, CRC	Completed CT/RT	35	36	56.9 ± 9.9/56.4 ± 12.7	94.3/86.1	MBSR/group sessions/physician	8 weeks (1/week, 2 h)	FSI	Yes	ACC (PES; general Education)	NR
14	Kubo (2019), USA [34]	NR	Various	Undergoing or recently completed CT/targeted therapy or immunotherapy	54	43	59.3 ± 14.1/56.7 ± 14.7	62.3/76.7	Mindfulness program/website or mobile Application/NR	8 weeks (10–20 min/day)	BFI	No	Wait‐list	NR
15	Lengacher (2012), USA [37]	0‐III	BC	Treatment received (RT only or RT and CT)	41	43	58 ± 9.4	100/100	MBSR/group sessions/CR	6 weeks (1/week, 2 h)	MDASI	Yes	Usual care	NR
16	Lengacher (2016), USA [36]	0‐III	BC	Completed treatment	167	155	56.5 ± 10.2/57.6 ± 9.2	100/100	MBSR/group sessions/CR	6 weeks (1/week, 2 h)	FSI	Yes	Usual care	NR
17	Lengacher (2021), USA [35]	0‐III	BC	Lumpectomy/mastectomy/adjuvant RT/CT	165	155	NR	100/100	MBSR/group sessions/CR	6 weeks (1/week, 2 h)	FSI	No	Usual care	NR
18	Lengacher (2025), USA [38]	I‐III	BC	Completed CT or CT/RT	91	31	56.3 ± 11.4/59.5 ± 11.0	100/100	MBSR/group sessions/CR and social worker	6 weeks (1/week, 2 h)	PROMIS	No	Usual care	None
19	Liu (2019), China [43]	I‐IV	DTC	Post‐thyroidectomy, pre‐RIT	49	53	43.32 ± 10.99/42.38 ± 12.6	69.3/77.5	MBSR/group sessions/CR	8 weeks (1/week)	QLQ‐C30	No	Usual care	NR
20	Liu & Liu (2022), China [44]	I‐II	BC	Post‐mastectomy and Postoperative CT	68	68	NR	100/100	Mindfulness yoga training/group sessions/NR	8 weeks (1/week, 1.5 h)	RPFS‐CV	No	Routine care	Mild knee pain (1.4%), temporary lower LEW (1.4%)
21	Liu & Wang (2022), China [42]	0‐IV	BC	Undergoing CT	38	34	48.58 ± 8.48/51.41 ± 10.10	100/100	MBSR/group sessions, WeChat/nurse	8 weeks (1/week, 45 min)	BFI‐C	No	Routine care	NR
22	Metin (2019), Turkey [55]	I‐III	BC	Patient receiving the first dose of adjuvant paclitaxel	32	29	48.21 ± 10.23/52.86 ± 11.70	100/100	MM/group sessions, WeChat/nurse	12 weeks (20 min/each day)	BFI	Yes	Single time education	None
23	Park (2020), Japan [51]	0‐III	BC	Patients with BC who are undergoing treatment	38	36	53.21 ± 8.4/54.19 ± 9.27	100/100	MBCT/group sessions/CR, psychiatrists, nurses	8 weeks (1/week, 2 h)	BFI	No	Wait‐list	None
24	Rahmani (2014), Iran [48]	I‐III	BC	Patients with a duration of diagnosis longer than 1 month	12	12	43.25 ± 3.08/44.08 ± 3.28	100/100	MBSR/group sessions/CR	8 weeks (1/week, 2 h)	QLQ‐C30	No	No intervention	None
25	Reich (2017), USA [39]	0‐III	BC	Post‐treatment	152	145	56.6	100/100	MBSR/group sessions/CR	6 weeks (1/week, 2 h)	FSI	No	Usual care	NR
26	Sheikhzadeh (2021), Iran [49]	NR	Various	Patients diagnosed with cancer at least 6 months ago	20	20	35/37	89/70	MBCT/group sessions/CR	8 weeks (1.5/week, 2 h)	CFS	Yes	Wait‐list	NR
27	Speca (2000), Canada [50]	I ‐IV	Various	Patients who are undergoing or have completed treatment	61	48	54.9 ± 10.5/48.9 ± 13.2	85.2/70.8	MBSR/group sessions/NR	7 weeks (1.5 h/week)	POMS	No	Wait‐list	NR
28	van der Lee (2012), Netherlands [52]	NR	Various	Cancer survivors who completed curative treatment at least 1 year ago	59	24	53.1 ± 9.1/49.4 ± 11.0	86/78	MBCT/group sessions/therapists	9 weeks (1/week, 2.5 h and one 6 h session)	CIS	Yes	Wait‐list	NR
29	Wang (2022), China [45]	0‐IV	BC	Patients who are 1–24 months post‐surgery	51	52	45.37 ± 7.59/48.17 ± 8.05	100/100	iMBCR/web‐based/therapist	4 weeks (1/week, 1.5 h)	MDASI‐C	No	Usual care	NR

Abbreviations: ACC, active control condition; AOMC, augmented optimal medical care program; BC, breast cancer; BFI, Brief Fatigue Inventory; CFS, Cancer Fatigue Scale; CIS, Checklist Individual Strength; CR, clinical psychologists; CRC, colorectal cancer; CT, chemotherapy; DTC, differentiated thyroid cancer; EORTC‐QLQ‐C30, European Organization for Research and Treatment of Cancer Quality of Life Questionnaire; FACIT‐F, Functional Assessment of Chronic Illness Therapy‐Fatigue Scale; FSI, Fatigue Symptom Inventory; FSS, Fatigue Severity Scale Questionnaire; HC, hematological cancer; HSCT, hematopoietic stem cell transplantation; LEW, lower extremity weakness; MAPs, mindful awareness practices; MBAT, mindfulness‐based art therapy; MBCT, mindfulness‐based cognitive therapy; MBI, mindfulness‐based intervention; MBSR, mindfulness‐based stress reduction; MDASI, M.D. Anderson Symptom Inventory; MFSI‐SF, Multidimensional Fatigue Scale Inventory‐Short Form; MM, mindfulness meditation;NR, not reported; PES, psychoeducation/support; POMS, Profile of Mood States; PROMIS, Patient‐Reported Outcomes Measurement Information System; QLQ‐C30, QoL Questionnaire Core 30 items; RIT, receiving radioactive iodine therapy; RPFS‐CV, Piper Fatigue Assessment Scale (Chinese version); RT, radiotherapy.

### 3.3 Risk of Bias

**FIGURE 2:**
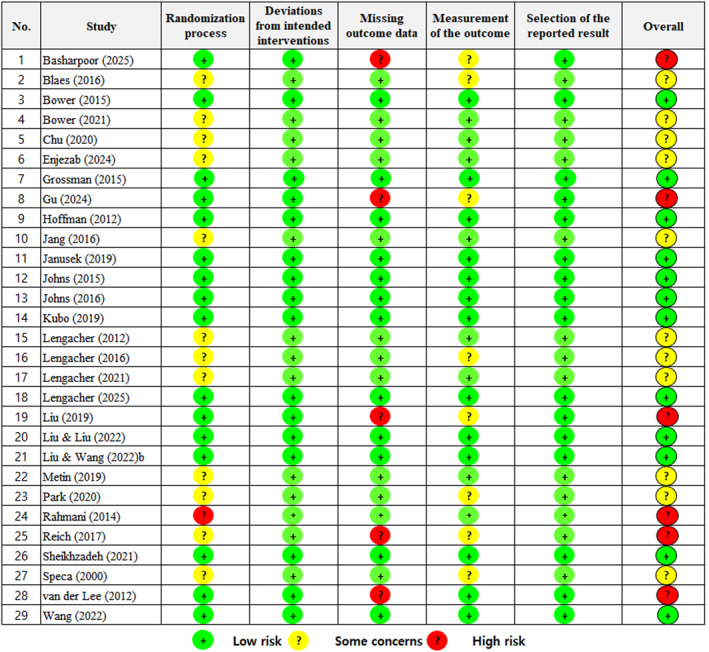
Risk‐of‐bias summary according to the revised Cochrane risk‐of‐bias 2.0 tool for randomized trials.

The risk‐of‐bias assessment of the 29 studies indicated low risk in 12 studies (41.4%); some concerns in 11 studies (37.9%); and high risk in 6 studies (20.7%). The detailed evaluation by domain was as follows. For the randomization process, 12 studies (41.4%) were rated as having some concerns because of insufficient information about whether the allocation sequence was concealed until participants were enrolled and assigned to interventions. One study (3.4%) was rated as high risk; in this study, although the allocation sequence was assumed to be random, no specific description was provided and no information about allocation concealment was reported. For deviations from intended interventions, all studies were judged as having low risk of bias. For missing outcome data, 5 studies (17.2%) were rated as high risk due to a high level of missing data, with outcome data unavailable for nearly all randomized participants. For the outcome measurement, 8 studies (27.6%) were assessed as having some concerns owing to insufficient information about whether outcome assessors were blinded to the intervention assignment, which could have influenced outcome evaluation. Finally, for the selection of reported results, all studies were rated as having low risk of bias (Figure 2).

### 3.4 Efficacy of Mindfulness‐Based Interventions for Reducing Cancer‐Related Fatigue

**FIGURE 3:**
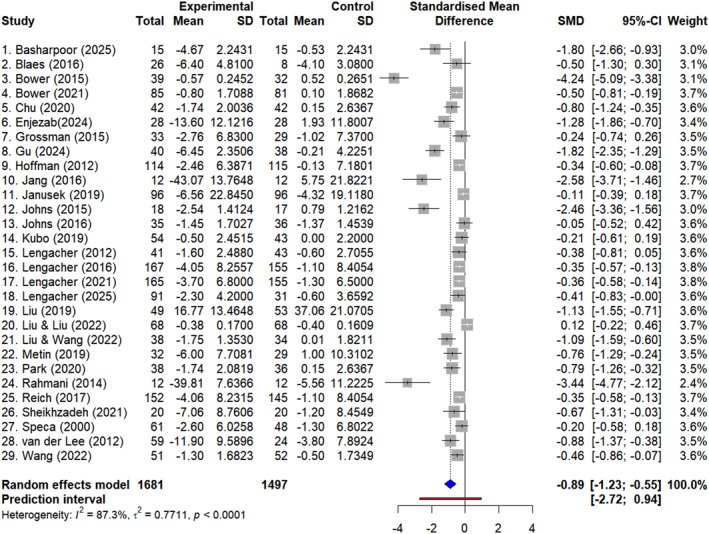
Forest plots: effect of mindfulness‐based interventions on fatigue. CI: confidence interval; SMD: standardized mean difference.

This synthesis included data from 29 RCTs with 3178 participants. The meta‐analytic findings demonstrated a significant reduction in fatigue following MBIs (SMD = −0.89; 95% CI: −1.23 to −0.55; *p* < 0.001; prediction interval: −2.05 to 0.27; Figure 3), with the negative SMD indicating greater fatigue reduction in the MBI groups when compared with control conditions. However, substantial heterogeneity was observed across studies (*I* ^2^ = 87.3%).

### 3.5 Subgroup and Moderator Analyses

Given the substantial heterogeneity observed in the overall analysis (*I* ^2^ = 87.3%), subgroup analyses and meta‐regressions were conducted to identify potential sources of variability. Specifically, subgroup analyses were performed for categorical moderators, including cancer type, treatment status, program type, intervention duration, delivery method, and MBI instructor. Differences in the effect sizes across these subgroups were not significant, suggesting that these variables were not major contributors to the observed heterogeneity.

Meta‐regression analysis for continuous moderators revealed that mean age and the proportion of female participants were not significant moderators of effect size heterogeneity. However, sample size accounted for 17.64% of the total heterogeneity, suggesting that differences in sample size partially explained the observed heterogeneity across studies (Table 2).

**TABLE 2:** Subgroup and moderator analysis.

Categorical moderators	k	SMD	95% CI	*I* ^2^ (%)	Between groups	
					Q_b_ (df)	*P*_b_
Cancer type						
Breast cancer	2	−0.90	−1.33, −0.46	88.5	1.1 (2)	0.603
Various	18	−0.73	−1.35, −0.11	78.8		
Others	9	−1.46	−2.75, −0.17	74.9		
Treatment status						
Complete treatment	12	−1.10	−1.63, −0.56	91.0	1.36 (2)	0.505
Under treatment	16	−0.78	−1.24, −0.32	83.8		
Mixed	1	−0.20	−2.00, 1.60	0.0		
Type of program						
MBSR	15	−0.84	−1.33, −0.34	86.1	0.37 (3)	0.945
MBCR	3	−0.89	−2.03, 0.24	74.1		
MBCT	4	−0.78	−1.74, 0.17	0.0		
Other MBIs	7	−1.09	−1.83, −0.35	94.2		
Duration of intervention						
< 8 weeks	10	−0.89	−1.46, −0.31	90.9	0.01 (2)	0.994
≥ 8 weeks	19	−0.89	−1.32, −0.46	84.6		
Delivery method						
In‐person (group sessions)	23	−0.88	−1.282, −0.48	87.7	0.01 (2)	0.994
Online (web or app)	4	−0.93	−1.87, 0.04	88.3		
Combination (in‐person & online)	2	−0.93	−2.26, 0.40	0.0		
MBI instructor						
Certified instructor	8	−0.98	−1.67, −0.28	77.9	0.77 (4)	0.941
Clinical psychologist	8	−0.74	−1.42, −0.05	37.0		
Nurse	2	−0.93	−2.30, 0.44	0.0		
Others	6	−0.75	−1.55, 0.03	76.7		
NR	5	−1.17	−2.04, −0.31	96.5		

**Continuous moderators**	**K**	**QM**	*P*	**Β**	**SE**	R^2^
Mean age	26	3.21	0.073	0.053	0.029	9.32
Sample size	29	6.40	0.011	0.009	0.003	17.64
Female percentage	29	1.39	0.237	−0.016	0.013	0.00

Abbreviations: CI, confidence interval; df, degrees of freedom; MBCR, mindfulness‐based cancer recovery; MBCT, mindfulness‐based cognitive therapy; MBSR, mindfulness‐based stress reduction; NR, not reported; P_b_, P between groups; Q_b_, Q between groups; QM, Q moderator.

### 3.6 Publication Bias

**FIGURE 4:**
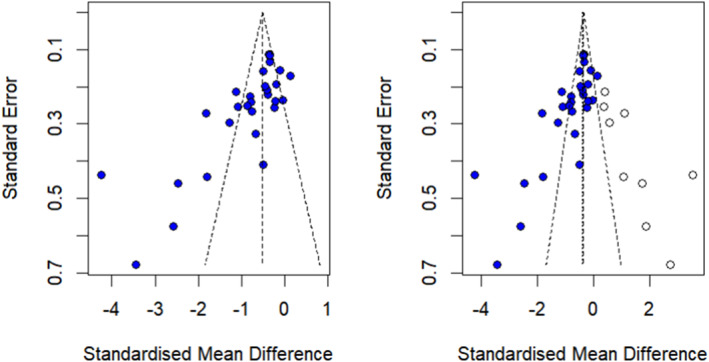
(A) Forest plot of the standard error by effect size for fatigue; (B) Funnel plot after including other studies via the trim‐and‐fill method.

Analysis of the fatigue outcome indicated potential publication bias, evidenced by funnel plot asymmetry (i.e., an empty region in the lower‐right section of the funnel plot; Figure 4A). Egger's regression test confirmed significant publication bias (*t* = −5.01, df = 27, *p* < 0.001). The trim‐and‐fill method imputed nine additional studies, reducing the SMD from −0.89 (95% CI: −1.23 to −0.55) to −0.38 (95% CI: −0.63 to −0.13) after adjustment (Figure 4B). Although the effect size was attenuated, MBIs remained significantly more effective than controls for reducing fatigue.

### 3.7 Sensitivity Analysis

The Baujat plot identified four studies [30, 32, 41, 44] as having the largest impact on the overall effect size (Supporting Information S1: Figure S1). Among these, Bower, et al. [30] exerted the greatest influence. Excluding this study reduced the SMD from −0.89 (95% CI: −1.23 to −0.55) to −0.74 (95% CI: −0.98 to −0.50) (Supporting Information S1: Figure S2), yet the overall effect remained significant. This indicated the robustness and stability of the findings. Additionally, a sensitivity analysis was conducted by excluding six studies [39, 41, 43, 46, 48, 52], assessed as high overall risk of bias. The pooled effect size remained largely unchanged, ranging from SMD = −0.82 to −0.91, confirming the robustness of the findings even when excluding high‐risk studies.

### 3.8 GRADE Quality of Evidence

The overall quality of evidence for the effect of MBIs on CRF was rated as moderate. Substantial heterogeneity was observed among included studies (*I* ^2^ = 87.3%), and potential sources of heterogeneity could not be adequately explained, suggesting possible inconsistencies (serious). Publication bias was also detected, and trim‐and‐fill adjustment substantially reduced the SMD. This indicated that the overall effect may have been overestimated (strongly suspected). Other domains, including the risk of bias, indirectness, and imprecision, were not considered serious (Supporting Information S1: Table S6).

## 4 Discussion

### 4.1 Interpretation of the Results and Comparison With Previous Studies

This comprehensive systematic review and meta‐analysis evaluated the efficacy of MBIs in alleviating CRF, a prevalent and debilitating symptom among patients with cancer. By synthesizing data from 29 RCTs involving 3178 participants, MBIs were found to be associated with significant reductions in fatigue. The observed effects of MBIs may be explained by several behavioral and psychological mechanisms, including improved attentional regulation, increased acceptance of bodily sensations, and reduced maladaptive stress responses. In addition, MBIs may contribute to CRF alleviation through physiological pathways, such as reduced cortisol secretion via modulation of the hypothalamic–pituitary–adrenal (HPA) axis [57], attenuated inflammatory responses (reflected by decreases in markers such as C‐reactive protein and interleukin‐6) [58], and enhanced autonomic regulation (as indicated by increased heart rate variability) [59]. However, substantial heterogeneity, moderate GRADE certainty of evidence, and evidence of strong publication bias limit confidence in these findings. Overall, MBIs represent a promising nonpharmacologic approach for managing CRF, although conclusions regarding their efficacy warrant cautious interpretation.

Our findings are consistent with those of Xu, et al. [60] who reported a large effect of online MBIs in reducing fatigue among patients with cancer. However, their review was limited to online delivery formats and included only two studies, restricting the reliability of their conclusions. In contrast, our study synthesized evidence from 29 RCTs encompassing both online and in‐person interventions, yielding more comprehensive and robust results. Similarly, Xie, et al. [18] reported a large effect size for MBIs in reducing fatigue, consistent with our findings. However, their review focused exclusively on MBSR and primarily included studies from Chinese databases, limiting generalizability. In comparison, our review encompassed a broader range of MBI formats and studies from multiple countries, thereby enhancing external validity. Our findings also align with those of McCloy, et al. [15] although their study was limited to women with cancer and included quasi‐experimental designs.

Simultaneously, our findings also differ from those of several studies. For instance, Chayadi, et al. [17] reported a small effect size. However, their review included non‐RCTs, while the present study exclusively focused on RCTs, thereby reflecting a fundamental difference in study design. Similarly, Castanhel, Liberali [16] and Chang, et al. [61] only reported small effect sizes, but each included two and four studies, respectively, and focused exclusively on patients with breast cancer. The limited number of studies and restricted population group likely contributed to these differences.

Additionally, several previous reviews [13, 19, 20, 21, 22] reported only moderate effect sizes, limited by the small number of studies (ranging from five to eight), restricting their ability to perform essential analyses such as exploring heterogeneity, assessing publication bias, or conducting sensitivity tests. He, et al. [20] focused solely on MBSR and did not account for other types of MBIs, Schell, et al. [22] only included patients with breast cancer, Zhang, et al. [13] only included non‐RCTs and restricted their population to patients with breast cancer, all limiting generalizability and internal validity.

In addition, some studies reported a nonsignificant effect of MBIs on fatigue. For example, two studies [62, 63] found no significant effects; however, each included only a limited number of studies (two and six, respectively) and focused exclusively on patients with breast cancer receiving MBSR. These methodological limitations reduce the generalizability and reliability of the conclusions. Overall, our findings differ from previous reviews due to variations in the study design, number of trials, and participant populations. By applying strict inclusion criteria, including only high‐quality RCTs, and incorporating a diverse cancer population, this study provides more robust and reliable evidence on the efficacy of MBIs in reducing CRF among patients with cancer. However, while the findings suggest that MBIs may contribute to reductions in CRF across various contexts, the substantial heterogeneity resulting from the diverse MBI formats, fatigue measures targeting different dimensions, and variations in cancer types and disease stages limits the ability to draw definitive conclusions based on the current evidence.

### 4.2 Strengths and Limitations

This study has the following strengths. First, by including 29 RCTs involving 3178 cancer patients, this meta‐analysis provides a relatively comprehensive synthesis of the available evidence, which may contribute to enhancing the reliability of the findings. Second, the inclusion of diverse cancer populations and MBI formats may help improve the external validity of the results. Third, unlike previous studies that primarily focused on face‐to‐face interventions, this study included both online and face‐to‐face delivery modes, allowing for a broader assessment of potential intervention effects across delivery modalities. Fourth, rigorous inclusion criteria were applied to include only RCTs and exclude studies in which the control group received active interventions similar to MBIs, which may help provide a more accurate estimation of intervention effects.

However, the study also has some limitations. First, substantial heterogeneity was observed among the studies, and although subgroup and meta‐regression analyses explored potential moderators, other factors may also have influenced differences in the observed effect size. Second, most included studies were conducted among female breast cancer patients, limiting the generalizability of the findings. In particular, given that the evidence is mainl**y** based on this specific population, it should not be interpreted as a definitive clinical recommendation. Therefore, additional high‐quality RCTs with adequate sample sizes across diverse cancer populations are needed to evaluate potential differences in MBI effects by cancer type. Third, although publication bias was explored and adjusted using methods such as the trim‐and‐fill, it could not be completely ruled out due to the limited number of unpublished studies identified. Fourth, despite a comprehensive search conducted without language restrictions, only English‐language studies were included, which may have introduced language bias. Fifth, due to the lack of consensus on the relative importance of the different dimensions of cancer‐related fatigue, no a priori outcome hierarchy or analytic priorities were specified, and all relevant outcomes were synthesized. While this increased inclusiveness, it also allowed for potential post hoc interpretation, further limiting the causal interpretability of the pooled estimates. Finally, although a more comprehensive evaluation was attempted by including diverse cancer populations, MBIs with differing clinical focuses, and various fatigue measurement instruments, this may have introduced additional clinical heterogeneity, which could limit the interpretation and generalizability of the findings.

### 4.3 Implications

This study suggests that MBIs may be considered potentially viable nonpharmacological interventions for the management of CRF. In particular, for patients who experience a high treatment burden or have concerns regarding medication‐related side effects, MBIs may serve as an adjunctive option. However, the substantial between‐study heterogeneity, along with evidence of small‐study effects and publication bias, indicates that the pooled effect estimate is unlikely to represent a stable or consistent clinical benefit across diverse settings. In particular, the wide prediction interval, which includes the null effect, suggests that in certain clinical contexts, MBIs may not produce a meaningful benefit for CRF. Differences in patient characteristics, cancer type and stage, intervention content, delivery format, and study size are likely to have contributed to the observed inconsistency in effects. Accordingly, the application of MBIs should be guided by cautious clinical judgment, taking into account individual symptom severity, treatment phase, and patient preferences. Any clinical implementation should proceed with explicit recognition of the uncertainty, heterogeneity, and limited generalizability inherent in the current evidence base.

## 5 Conclusion

This meta‐analysis suggests that MBIs may be associated with reductions in CRF; however, the substantial between‐study heterogeneity and the wide prediction interval indicate that these findings are unlikely to represent a stable or consistent clinical benefit across settings. Moreover, the moderate certainty of the evidence according to the GRADE framework and the indication of strong publication bias further limit confidence in these results. Therefore, the pooled estimates should not be interpreted as definitive evidence of the efficacy of MBIs but rather as an average effect estimate across heterogeneous interventions, outcome measures, and cancer populations. Future research should include large‐scale, methodologically rigorous RCTs using standardized CRF measures to minimize biases from small studies and enhance the reliability, precision, and clinical applicability of the evidence.

## Author Contributions

**Jihyun Lee:** data selection and extraction, methodology, writing–review and editing, and supervision. **Myoungsuk Kim:** conceptualization, data selection and extraction, statistical analysis, methodology, data curation, visualization, writing–original draft, writing–review and editing, and supervision. All the authors have read and approved the final version of this manuscript.

## Funding

The authors have nothing to report.

## Ethics Statement

An ethics statement is not applicable because this study was based exclusively on published literature.

## Conflicts of Interest

The authors declare no conflicts of interest.

## Supporting information


Supporting Information S1

## Data Availability

The data that support the findings of this study are available from the corresponding author upon reasonable request.
