# Supplementary material for: Efficacy of Mindfulness‐Based Interventions for Reducing Cancer‐Related Fatigue: A Systematic Review and Meta‐Analysis
Source: Psychooncology. 2026 Mar 23;35(3):e70435. doi: 10.1002/pon.70435 (PMC13009704; doi:10.1002/pon.70435)
Supplement: Supplementary file 1 — Supporting Information S1 [file PON-35-e70435-s001.docx]

**Supplementary Materials**

**Supplemental Table S1. PubMed**

| **Search #** | **MeSH Terms and Key Words** | **Articles Revealed** |
| --- | --- | --- |
| #1 | "Neoplasms"[MeSH Terms] OR "Neoplasms"[All Fields] OR "Tumors"[All Fields] OR "Neoplasia"[All Fields] OR "Neoplasias"[All Fields] OR "Neoplasm"[All Fields] OR "Tumor"[All Fields] OR "Cancer"[All Fields] OR "Cancers"[All Fields] OR "Malignant Neoplasm"[All Fields] OR "Malignancy"[All Fields] OR "Malignancies"[All Fields] OR "Malignant Neoplasms"[All Fields] OR "neoplasm malignant"[All Fields] OR "neoplasms malignant"[All Fields] OR "Benign Neoplasms"[All Fields] OR "neoplasms benign"[All Fields] OR "neoplasm benign"[All Fields] OR "Benign Neoplasm"[All Fields] | 5,997,636 |
| #2 | "Mindfulness"[MeSH Terms] OR "Mindfulness"[All Fields] OR "Mindfulness-based intervention"[All Fields] OR "mindfulness based"[All Fields] OR "mindfulness based"[All Fields] OR "MBSR"[All Fields] OR "MBCT"[All Fields] OR "mindfulness based stress reduction"[All Fields] OR "mindfulness based stress reduction"[All Fields] OR "mindfulness based cognitive therapy"[All Fields] OR "mindfulness based cognitive therapy"[All Fields] OR "Body-mind"[All Fields] OR "Mind-body"[All Fields] | 26,190 |
| #3 | "Fatigue"[MeSH Terms] OR "Fatigue"[All Fields] OR "Lassitude"[All Fields] | 160,770 |
| #4 | "Randomized Controlled Trial"[All Fields] OR "Randomised Controlled Trial"[All Fields] OR "Randomized"[All Fields] OR "Randomised"[All Fields] OR "Randomization"[All Fields] OR "Randomisation"[All Fields] | 1,222,647 |
| #5 | #1 AND #2 AND #3 AND #4 | 231 |

**Supplemental Table S2. Web of Science**

| **Search #** | **MeSH Terms and Key Words** | **Articles Revealed** |
| --- | --- | --- |
| #1 | ALL=("Neoplasms"[Mesh]) OR (“Neoplasms” OR "Tumors" OR "Neoplasia" OR "Neoplasias" OR "Neoplasm" OR "Tumor" OR "Cancer" OR "Cancers" OR "Malignant Neoplasm" OR "Malignancy" OR "Malignancies" OR "Malignant Neoplasms" OR "Neoplasm, Malignant" OR "Neoplasms, Malignant" OR "Benign Neoplasms" OR "Neoplasms, Benign" OR "Neoplasm, Benign" OR "Benign Neoplasm") | 4,409,735 |
| #2 | ALL=("Mindfulness"[Mesh]) OR (“Mindfulness” OR "Mindfulness-based intervention" OR "Mindfulness-based" OR "Mindfulness based" OR "MBSR" OR "MBCT" OR "Mindfulness based stress reduction" OR "Mindfulness-based stress reduction" OR "Mindfulness based cognitive therapy" OR "Mindfulness-based cognitive therapy" OR "Body-mind" OR "Mind-body") | 42,355 |
| #3 | ALL=("Fatigue"[Mesh]) OR “Fatigue” OR “Lassitude”) | 325,033 |
| #4 | ALL=("Randomized Controlled Trial" OR "Randomised Controlled Trial" OR "Randomized" OR "Randomised" OR "Randomization" OR "Randomisation") | 1,099,061 |
| #5 | #1 AND #2 AND #3 AND #4 | 320 |

**Supplemental Table S3.** Cochrane Library

| **Search #** | **MeSH Terms and Key Words** | **Articles Revealed** |
| --- | --- | --- |
| #1 | MeSH descriptor: [Neoplasms] explode all trees OR( “Neoplasms” OR "Tumors" OR "Neoplasia" OR "Neoplasias" OR "Neoplasm" OR "Tumor" OR "Cancer" OR "Cancers" OR "Malignant Neoplasm" OR "Malignancy" OR "Malignancies" OR "Malignant Neoplasms" OR "Neoplasm, Malignant" OR "Neoplasms, Malignant" OR "Benign Neoplasms" OR "Neoplasms, Benign" OR "Neoplasm, Benign" OR "Benign Neoplasm") | **307,927** |
| #2 | MeSH descriptor: [Mindfulness] explode all trees OR (“Mindfulness” OR "Mindfulness-based intervention" OR "Mindfulness-based" OR "Mindfulness based" OR "MBSR" OR "MBCT" OR "Mindfulness based stress reduction" OR "Mindfulness-based stress reduction" OR "Mindfulness based cognitive therapy" OR "Mindfulness-based cognitive therapy" OR "Body-mind" OR "Mind-body") | **12,983** |
| #3 | MeSH descriptor: [Fatigue] explode all trees OR (“Lassitude”) | **6,357** |
| #4 | MeSH descriptor: [Randomized Controlled Trial] explode all trees OR (“Randomized Controlled Trial” OR “Randomized” OR “Randomised” OR “Randomization” OR “Randomisation”) | **1,353,972** |
| #5 | #1 AND #2 AND #3 AND #4 | **84** |

**Supplemental Table S4.** CINAHL with Full Text

| **Search #** | **Subject Headings (MH) and Key Words** | **Articles Revealed** |
| --- | --- | --- |
| S1 | (MH " Neoplasms") OR TI (“Neoplasms” OR "Tumors" OR "Neoplasia" OR "Neoplasias" OR "Neoplasm" OR "Tumor" OR "Cancer" OR "Cancers" OR "Malignant Neoplasm" OR "Malignancy" OR "Malignancies" OR "Malignant Neoplasms" OR "Neoplasm, Malignant" OR "Neoplasms, Malignant" OR "Benign Neoplasms" OR "Neoplasms, Benign" OR "Neoplasm, Benign" OR "Benign Neoplasm") | 499,718 |
| S2 | (MH " Mindfulness") OR TI (“Mindfulness” OR "Mindfulness-based intervention" OR "Mindfulness-based" OR "Mindfulness based" OR "MBSR" OR "MBCT" OR "Mindfulness based stress reduction" OR "Mindfulness-based stress reduction" OR "Mindfulness based cognitive therapy" OR "Mindfulness-based cognitive therapy" OR "Body-mind" OR "Mind-body") | 13,433 |
| S3 | (MH " Fatigue ") OR TI (“Lassitude” ) | 24,424 |
| S4 | (PT randomized controlled trials) OR TI ("Randomized Controlled Trial" OR "Randomised Controlled Trial" OR "Randomized" OR "Randomised" OR "Randomization" OR "Randomisation") | 167,851 |
| S5 | S1 AND S2 AND S3 AND S4 | **15** |

**Supplemental Table S5.** Embase

| Search # | Emtree Terms and Key Words | Articles Revealed |
| --- | --- | --- |
| #1 | 'malignant neoplasm'/exp OR 'neoplasms' OR 'tumors' OR 'neoplasia' OR 'neoplasias' OR 'neoplasm' OR 'tumor' OR 'cancer' OR 'cancers' OR 'malignant neoplasm' OR 'malignancy' OR 'malignancies' OR 'malignant neoplasms' OR 'neoplasm, malignant' OR 'neoplasms, malignant' OR 'benign neoplasms' OR 'neoplasms, benign' OR 'neoplasm, benign' OR 'benign neoplasm' | 8,466,339 |
| #2 | 'mindfulness'/exp OR 'mindfulness' OR 'mindfulness-based intervention' OR 'mindfulness-based' OR 'mindfulness based' OR 'mbsr' OR 'mbct' OR 'mindfulness based stress reduction' OR 'mindfulness-based stress reduction' OR 'mindfulness based cognitive therapy' OR 'mindfulness-based cognitive therapy' OR 'body-mind' OR 'mind-body' | 37,961 |
| #3 | 'fatigue'/exp OR  'lassitude' | 385,280 |
| 4 | 'randomized controlled trial'/exp OR 'randomized controlled trial' OR 'randomised controlled trial' OR 'randomized' OR 'randomised' OR 'randomization' OR 'randomisation' | 1,980,786 |
| #6 | #1 and #2 and #3 and #4 | 487 |

**Supplemental Table S6. GRADE evidence profile and summary of findings table for the outcomes**

| **Outcome** | **Certainty assessment** | | | | | | | **Anticipated absolute effects (95% CI)** |
| --- | --- | --- | --- | --- | --- | --- | --- | --- |
|  | **№ of participants (studies)** | **Risk of bias** | **Inconsistency** | **Indirectness** | **Imprecision** | **Publication bias** | **Overall certainty of evidence** |  |
| **Fatigue** | 3,125 (29 RCTs) | not serious | serious | not serious | not serious | strongly  suspected | ⨁⨁⨁◯ Moderate ^a,b^ | SMD -0.89 (-1.23 to -0.55) |
| **Abbreviations:** CI, confidence interval; GRADE, grading of recommendations, assessment, development, and evaluation; RCT, randomized controlled trial; SMD, standardized mean difference | | | | | | | | |
| **GRADE Working Group grades of evidence**  **High certainty:** We are very confident that the true effect lies close to the estimated effect.  **Moderate certainty:** We are moderately confident in the effect estimate: the true effect is likely to be close to the estimated effect, but there is a possibility that it is substantially different.  **Low certainty:** Our confidence in the effect estimate is limited: the true effect may be substantially different from the estimated effect.  **Very low certainty:** We have very little confidence in the effect estimate: the true effect is likely to be substantially different from the estimated effect.  Explanations  ^a^Considerable heterogeneity is observed among the studies (I² =87.2%), and although potential sources of heterogeneity are explored, they are not adequately explained, suggesting possible inconsistency.  ^b^Evidence of publication bias is detected, and adjustment using the trim-and-fill method results in a substantial reduction in the standardized mean difference (SMD), suggesting that the overall effect may have been somewhat overestimated. | | | | | | | | |


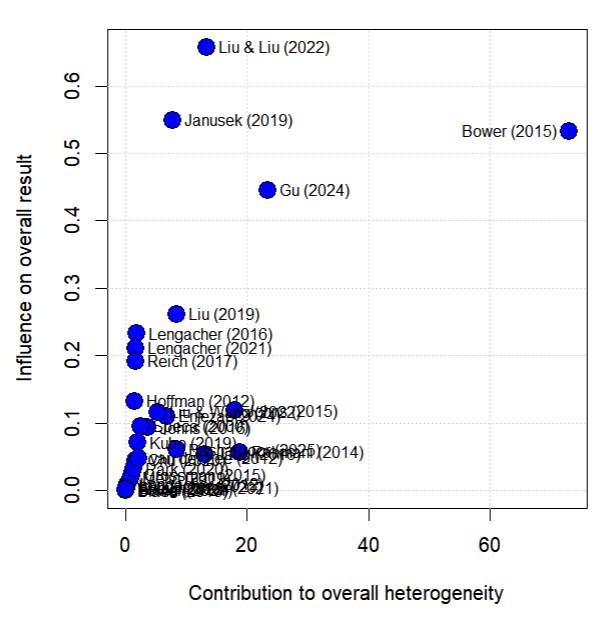


**Supplemental Figure S1.** Baujat plot on fatigue


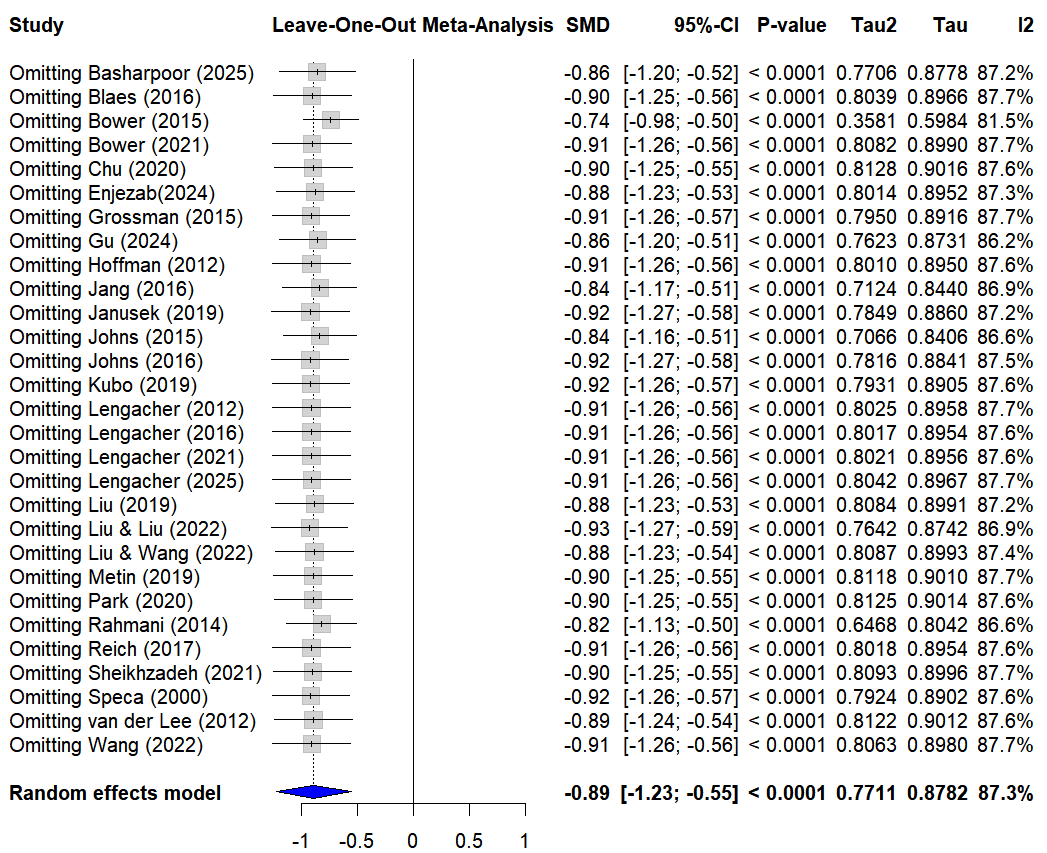


**Supplemental Figure S2.** Forest plot of sensitivity analysis
